# Genetic variability within and among *Haemonchus contortus* isolates from goats and sheep in China

**DOI:** 10.1186/1756-3305-6-279

**Published:** 2013-09-25

**Authors:** Fanyuan Yin, Robin B Gasser, Facai Li, Min Bao, Weiyi Huang, Fengcai Zou, Guanghui Zhao, Chunren Wang, Xin Yang, Yanqin Zhou, Junlong Zhao, Rui Fang, Min Hu

**Affiliations:** 1 State Key Laboratory of Agricultural Microbiology, College of Veterinary Medicine, Huazhong Agricultural University, Wuhan 430070, China; 2 Faculty of Veterinary Science, The University of Melbourne, Parkville, Victoria 3010, Australia; 3 College of Animal Sciences and Veterinary Medicine, Liaoning Medical College, Jingzhou 121000, China; 4 Department of Veterinary Medicine, College of Animal Science and Technology, Guangxi University, Nanning 530004, China; 5 College of Animal Science and Technology, Yunnan Agricultural University, Kunming 650201 China; 6 College of Veterinary Medicine, Northwest A&F University, Yangling 712100, China; 7 College of Animal Science and Veterinary Medicine, Heilongjiang Bayi Agricultural University, Daqing 163319, China

**Keywords:** *Haemonchus contortus*, Genetic variation, ITS-2, *nad*4, China

## Abstract

**Background:**

*Haemonchus contortus* (order Strongylida) is a common parasitic nematode infecting small ruminants and causing significant economic losses worldwide. Knowledge of genetic variation within and among *H. contortus* populations can provide a foundation for understanding transmission patterns, the spread of drug resistance alleles and might assist in the control of haemonchosis.

**Methods:**

152 *H. contortus* individual adult worms were collected from seven different geographical regions in China. The second internal transcribed spacer (ITS-2) of the nuclear ribosomal DNA and mitochondrial nicotinamide dehydrogenase subunit 4 gene (*nad*4) were amplified by polymerase chain reaction (PCR) and sequenced directly. The sequence variations and population genetic diversities were determined.

**Results:**

Nucleotide sequence analyses revealed 18 genotypes (ITS-2) and 142 haplotypes (*nad*4) among the 152 worms, with nucleotide diversities of 2.6% and 0.027, respectively, consistent with previous reports from other countries, including Australia, Brazil, Germany, Italy, Malaysia, Sweden, the USA and Yemen. Population genetic analyses revealed that 92.4% of nucleotide variation was partitioned within populations; there was no genetic differentiation but a high gene flow among Chinese populations; some degree of genetic differentiation was inferred between some specimens from China and those from other countries.

**Conclusions:**

This is the first study of genetic variation within *H. contortus* in China. The results revealed high within-population variations, low genetic differentiation and high gene flow among different populations of *H. contortus* in China. The present results could have implications for studying the epidemiology and ecology of *H. contortus* in China.

## Background

*Haemonchus contortus* is a trichostrongyloid nematode and one of the major pathogens affecting small ruminants worldwide [1]. The adult female of this species produces large numbers of eggs, which are excreted in hosts’ faeces. The eggs hatch on pasture and continue to develop under moist conditions to third-stage larvae (L3s). L3s are then ingested by a suitable host animal and eventually establish as dioecious, adult worms [2]. The blood-feeding activity of adults causes anaemia, oedema, diarrhoea and even death [3], consequently causing serious production and economic losses, particularly in tropical and temperate regions of the world [4].

Population genetic studies of *H. contortus* in the USA have shown that this species exhibits high within population variation and low genetic differentiation within continuous geographical regions, likely ascribing to high gene flow influenced by host movement [5,6]. However, strong barriers to gene flow have been observed on a global scale, which appear to be attributed to poor dispersal ability of the parasite and restricted opportunities for host movement across continents [7]. Other research, focused on domestic and wild animals, has found high genetic variation and relatively low host specificity for *H. contortus* within Brazil and Italy [8,9].

Population genetic studies of *H. contortus* have been conducted in a wide range of geographical regions of the world, including Australia, Brazil, Europe, Malaysia, and the USA [5,7-12]. However, surprisingly, nothing is known about genetic variability within *H. contortus* in China, in spite of its endemic status and economic impact in this country [13,14]. Therefore, in the present study, we explored genetic variation within and among seven populations of *H. contortus* from southwest, central and northeastern regions of China, employing the second internal transcribed spacer (ITS-2) of nuclear ribosomal DNA and the mitochondrial nicotinamide dehydrogenase subunit 4 (*nad*4) gene as markers.

## Methods

### Parasite material

**Figure 1:**
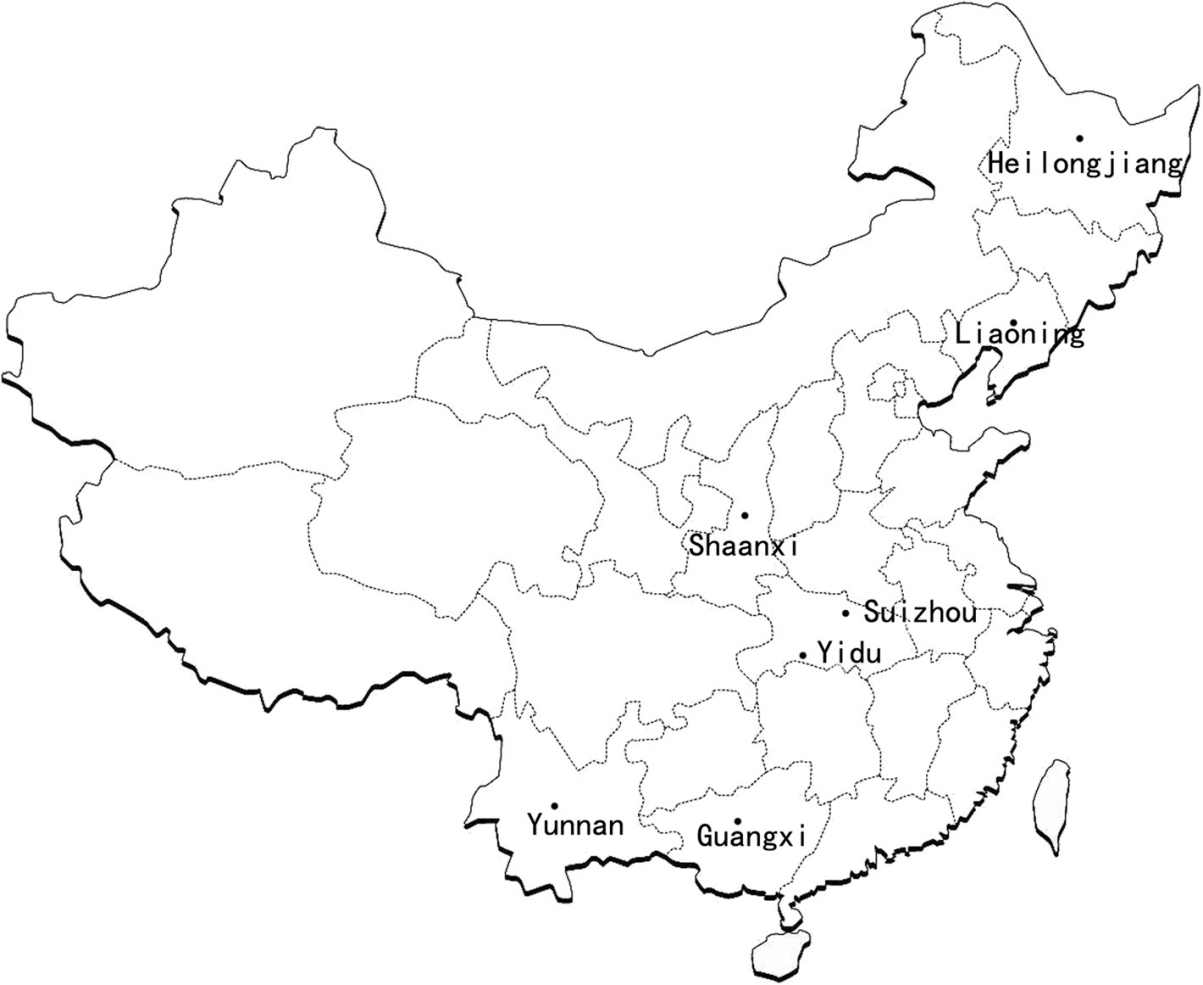
**Sampling sites.** Seven different geographical locations in China (longitudes and latitudes given in Table 1) at which adult *Haemonchus contortus* were collected from sheep or goats.

In total, 152 individual adult specimens of *H. contortus* were collected from the abomasa of slaughtered sheep or goats from seven geographical locations in tropical to subtropical climate zones and six provinces of China (Figure 1 and Table 1). Geographical locations were separated by distances of 370 to 4000 km. Samples from Liaoning and Heilongjiang were from sheep, whereas those from other regions were from goats. The adult specimens of *H. contortus* (17 to 24 per population) were washed extensively in physiological saline, stored in 70% ethanol and then sent to the College of Veterinary Medicine, Huazhong Agricultural University, Wuhan. Upon arrival, individual worms were identified morphologically, according to Lichtenfels *et al.* (1994) [15].

**Table 1:** **Sources of 152 adults of *Haemonchus contortus *representing seven different geographical locations in China, and the numbers of ITS-2 genotypes and *nad *4 haplotypes**

**Localities (longitude, latitude)**	**No. of ITS-2 amplicons sequenced**	**No. of ITS-2 genotypes**	**No. of*nad*4 amplicons sequenced**	**No. of*nad*4 haplotypes**
Suizhou	22	8	22	21
(112°43′E,31°19′N)				
Yidu	17	6	17	16
(111°47′E,30°39′N)				
Guangxi	23	5	23	23
(108°3′E,22°8′N)				
Yunnan	22	8	22	21
(102°42′E,25°04′N)				
Shaanxi	23	6	23	23
(107°40′E,33°39′N)				
Liaoning	24	8	24	23
(123°38′E,41°08′N)				
Heilongjiang	21	6	21	20
(124°19′E,45°46′N)				
Totals	152	18	152	142

### Isolation of genomic DNA

Total genomic DNA was isolated from individual worms using sodium dodecyl-sulfate/proteinase K treatment [16], followed by spin-column purification (Wizard™ DNA Clean-Up, Promega). DNA samples were stored at -20°C until use.

### PCR amplification and sequencing

ITS-2 (~350 bp) was amplified using the conserved primers: NC1 (5′-ACGTCTGGTTCAGGGTTGTT-3′) and NC2 (5′-TTAGTTTCTTTTCCTCCGCT-3′) [17]. A region (800 bp) of the *nad*4 gene was amplified by PCR using primer1-F (5′-GGATTTGGTCAGCAAATTGAA-3′) and primer2-R (5′-GCCTGCAAATGAATTAACA-3′) [7]. PCR (25 or 50 μl) was performed in 10 mM Tris–HCl, pH 8.3, 50 mM KCl, 4 mM MgCl_2_, 250 μM each of dNTP, 100 pmol of each primer and 1 U Taq polymerase (TaKaRa) under the following conditions: initial denaturation at 94°C for 5 min, followed by 30 cycles of denaturation at 94°C for 30 s, annealing at 55°C for 30 s and extension at 72°C for 1 min, with a final extension of 72°C for 5 min.

All PCR products were examined on agarose gels (1.5%) to verify that they represented single bands, column-purified (Wizard PCR-Preps, Promega) and then sequenced directly (BigDye Terminator v.3.1 cycle sequencing kit, Applied Biosystems) in an automated sequencer (PRISM3730, ABI) using appropriate forward and reverse primers (in separate reactions). Forward and reverse sequences were merged, consensus sequences determined and then deposited in the GenBank database.

### Data analysis

Sequences were aligned over a consensus length (231 bp for ITS-2 and 412 bp for *nad*4) using the program Clustal W within MEGA v.5.0 [18]. Pairwise comparisons were made with previously published sequences, and identities (%) calculated using the program BioEdit [19].

The phylogenetic analysis was conducted using the neighbour-joining (NJ), maximum parsimony (MP) and maximum likelihood (ML) methods, respectively, based on the Tamura -Nei model [18]. Confidence limits were assessed using bootstrap procedure with 1000 pseudo-replicates for NJ, MP and ML trees, and other settings were obtained using the default values in MEGA v.5.0 [18]. A 50% cut-off value was implemented for the consensus tree. The Collapse program online (http://sing.ei.uvigo.es/ALTER/) was used to define haplotype sequences. The diversity indices (Fst and Nst) were calculated using the program DnaSP5.1 [20] to evaluate degree of gene flow among populations. Tajima’s *D*[21] and Fu’s Fs [22] were also calculated to test neutrality using the same program (DnaSP 5.1) [20]. To estimate genetic diversity within and among populations (isolates), the hierarchical analysis of molecular variance (AMOVA) was performed using the Arlequin 3.1 package [23]. For this analysis, the data set was divided into three groups. Groups 1, 2 and 3 contained samples from Southwest (Guangxi, Yunnan), Central (Suizhou, Yidu, Shaanxi) and Northeast (Liaoning, Heilongjiang) China, respectively. The median joining (MJ) network [24] was drawn using the program Network v.4.6.1.1 to study of haplotype relationships. In addition, 176 *nad*4 sequences from previous studies [5,8,10] were retrieved from GenBank and used for comparisons. Information on geographical origins, accession numbers and parasite codes are given in Table 2.

**Table 2:** **Fst values between *Haemonchus contortus *populations of China and those from other four countries calculated from the *nad *4 sequence data**

**Geographical location**	**GenBank accession no.**	**No. of sequences**	**Fst**	**π**
Malaysia	HQ660255-HQ660308	54	0.05841	0.03545
Italy	AJ429793-AJ429809	17	0.14407	0.03263
Yemen	HQ660309-HQ660367	59	0.26710	0.03462
The USA	AF070736-AF070785	46	0.41294	0.02512
China	KC429944-KC430085	142	-	0.02745

A total of 176 *nad*4 sequences were retrieved from the GenBank database for comparison. The nucleotide diversity (π) within each population was also calculated.

## Results

### Sequences analyses

ITS-2 sequences were determined from 152 worms from seven different geographical locations in China (see Table 1). After sequence editing and alignment, a consensus length of 231 bp was obtained for all specimens. The analysis of the 152 ITS-2 sequences revealed 18 distinct genotypes (sequence data are publicly available under accession numbers KC415117-KC415134 for ITS-2), with sequence identities ranging from 97.4% to 100%, when compared with each other or with two reference sequences for *H. contortus* (GenBank accession nos. X78803 and EU084691; Table 3). These sequences were also compared with two publicly available ITS-2 sequences of *H. placei* (accession nos. X78812 and AJ577466), revealing the nucleotide identities of 96.1 to 98.2%. The alignment of all 18 ITS-2 sequences with the reference sequence X78803 revealed six substitutions at the nucleotide positions (10, 18, 21, 22, 123 and 196; see Additional file 1). These substitutions represented four transversions (one A < - > C, one G < - > C and two A < - > T substitutions) and two transitions (T < - > C). The nucleotide diversities and genotype diversities among the 18 ITS-2 sequences of *H. contortus* from China ranged from 0.0054 to 0.0084 and from 0.609 to 0.824, respectively (Table 4).

**Table 3:** **Pairwise identities (%) among the 18 ITS-2 sequences of *Haemonchus contorts *representing 152 adults of *H. contortus *from China using selected sequences for *H. contortus *and *H. placei *from the GenBank database**

**Sample code**	**1**	**2**	**3**	**4**	**5**	**6**	**7**	**8**	**9**	**10**	**11**	**12**	**13**	**14**	**15**	**16**	**17**	**18**	**19**	**20**	**21**	**22**
1-Hlj10 (KC415117)	-																					
2-Ln9 (KC415118)	99.5	-																				
3-SZ9 (KC415119)	99.5	99.1	-																			
4-Sz13 (KC415120)	98.7	98.2	99.1	-																		
5-Yd2 (KC415121)	99.1	98.7	99.5	99.5	-																	
6-Yn16 (KC415122)	99.1	98.7	98.7	99.5	99.1	-																
7-Yd14 (KC415123)	99.5	99.1	99.1	99.1	99.5	99.5	-															
8-SZ18 (KC415124)	99.5	99.1	99.1	99.1	98.7	99.5	99.1	-														
9-Sz22 (KC415125)	98.7	99.1	99.1	99.1	98.7	98.7	98.2	99.1	-													
10-Sz21 (KC415126)	99.1	99.5	99.5	98.7	99.1	98.2	98.7	98.7	99.5	-												
11-Sx7 (KC415127)	99.1	99.5	98.7	98.7	98.2	99.1	98.7	99.5	99.5	99.1	-											
12-YD7 (KC415128)	99.1	98.7	99.5	99.5	99.1	99.1	98.7	99.5	99.5	99.1	99.1	-										
13-Sz1 (KC415129)	98.2	98.7	98.7	98.7	98.2	98.2	97.8	98.7	99.5	99.1	99.1	99.1	-									
14-Sz12 (KC415130)	98.2	98.7	97.8	97.8	97.4	98.2	97.8	98.7	98.7	98.2	99.1	98.2	99.1	-								
15-Ln1 (KC415131)	97.8	98.2	98.2	98.2	97.8	97.8	97.4	98.2	99.1	98.7	98.7	98.7	99.5	99.5	-							
16-Gx3 (KC415132)	98.7	99.1	98.2	98.2	97.8	98.7	98.2	99.1	99.1	98.7	99.5	98.7	99.5	99.5	99.1	-						
17-Yn20 (KC415133)	98.7	98.2	99.1	99.1	98.7	98.7	98.2	99.1	99.1	98.7	98.7	99.5	99.5	98.7	99.1	99.1	-					
18-Gx7 (KC415134)	99.1	98.7	98.7	98.7	98.2	99.1	98.7	99.5	98.7	98.2	99.1	99.1	99.1	99.1	98.7	99.5	99.5	-				
19-*H.contortus* (X78803.1)	99.1	98.7	99.5	98.7	99.1	98.2	98.7	98.7	98.7	99.1	98.2	99.1	98.2	98.2	98.7	97.8	98.7	98.2	-			
20-*H.contortus* (EU084691.1)	100	99.5	99.5	98.7	99.1	99.1	99.5	99.5	98.7	99.1	99.1	99.1	98.2	98.2	97.8	98.7	98.7	99.1	99.1	-		
21-*H.placei* (X78812.1)	97.8	97.4	98.2	97.4	97.8	96.9	97.4	97.4	97.4	97.8	96.9	97.8	96.9	96.9	97.4	96.5	97.4	96.9	98.7	97.8	-	
*22-H.placei* (AJ577466.1)	97.4	96.9	97.8	96.9	97.4	96.5	96.9	96.9	96.9	97.4	96.5	97.4	96.5	96.5	96.9	96.1	96.9	96.5	98.2	97.4	99.5	-

**Table 4:** **Diversity and neutrality indices for different populations of *Haemonchus contortus *from seven different geographical locations in China, calculated from ITS-2 and *nad *4 nucleotide data sets**

	**ITS-2**		***nad*4**			
**Location**	**Gd**	**π**	**Hd**	**π**	***D***	**Fs**
SZ	0.649	0.0083	0.996	0.0212	-1.3764	-12.703
YD	0.824	0.0069	0.993	0.0205	-0.0442	-7.669
GX	0.609	0.0054	1.000	0.0226	-1.6408	-16.558
YN	0.654	0.0057	0.996	0.0369	-0.6390	-8.057
SX	0.708	0.0075	1.000	0.0178	-1.6031	-19.345
LN	0.656	0.0084	0.996	0.0263	-1.1294	-12.808
HLJ	0.824	0.0058	0.995	0.0196	-1.4605	-12.297

Abbreviations: haplotype diversity (Hd), nucleotide diversity (π), Tajima’s *D* (*D*), Fu’s Fs (Fs), genotype diversity (Gd). Locations: Suizhou (SZ), Yidu (YD), Guangxi (GX), Yunnan (YN), Shaanxi (SX), Liaoning (LN) and Heilongjiang (HLJ).

From the 152 *nad*4 amplicons, 142 distinct haplotypes (all with open reading frames) were defined (Table 1; sequence data are publicly available under accession numbers KC429944-KC430085 for *nad*4). Among 142 unique haplotypes, 73 parsimony informative sites were identified. Nucleotide diversities ranged from 0.0178 to 0.0369, and the haplotype diversity ranged from 0.993 to 1.000 for the seven distinct populations of *H. contortus* (Table 4). The highest nucleotide diversity estimated was for the *H. contortus* population from Yunnan. Tajima’s *D* and Fu’s Fs are also shown in Table 4; their low negative values implied no significant deviation from neutrality.

### Phylogenetic analysis

**Figure 2:**
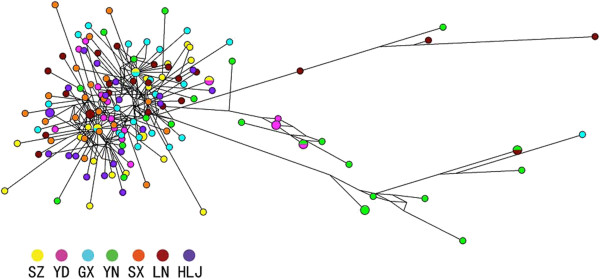
**The median joining network of 142 *nad*4 haplotypes representing 152 individuals of *Haemonchus contortus *from seven different geographical locations in China.** The different coloured dots represent haplotypes from the different populations/locations: SZ: Suizhou; YD: Yidu; GX: Guangxi; YN: Yunnan; SX: Shaanxi; LN: Liaoning; HLJ: Heilongjiang.

Trees were constructed using the *nad*4 sequence data for *H. contortus* from China, using an *nad*4 sequence of *H. placei* (accession no. AF070825) as the outgroup. The tree revealed a relatively continuous variation in genetic distance among haplotypes (Additional file 2). There was a random distribution of sequences representing different locations, with weak support (< 50%) for some nodes. There were no obvious boundaries among individuals in the phylogenetic trees, except that some individuals from the Yunnan population grouped with moderate nodal support (72%), with one sequence from Guangxi and one cluster contained one sequence from Yunnan and three sequences from Liaoning with strong nodal support (90%). Consistent with the results obtained for the NJ tree, analysis of the *nad*4 sequences using MP and ML methods, respectively, produced phylogenetic trees with similar topologies (not shown). Extending the analyses, to discern the relationships among 142 haplotypes, a parsimony network was constructed (Figure 2). The network profile showed that there was no preferential or distinct grouping of specimens according to geographical region, except for several individuals from Yunnan.

**Figure 3:**
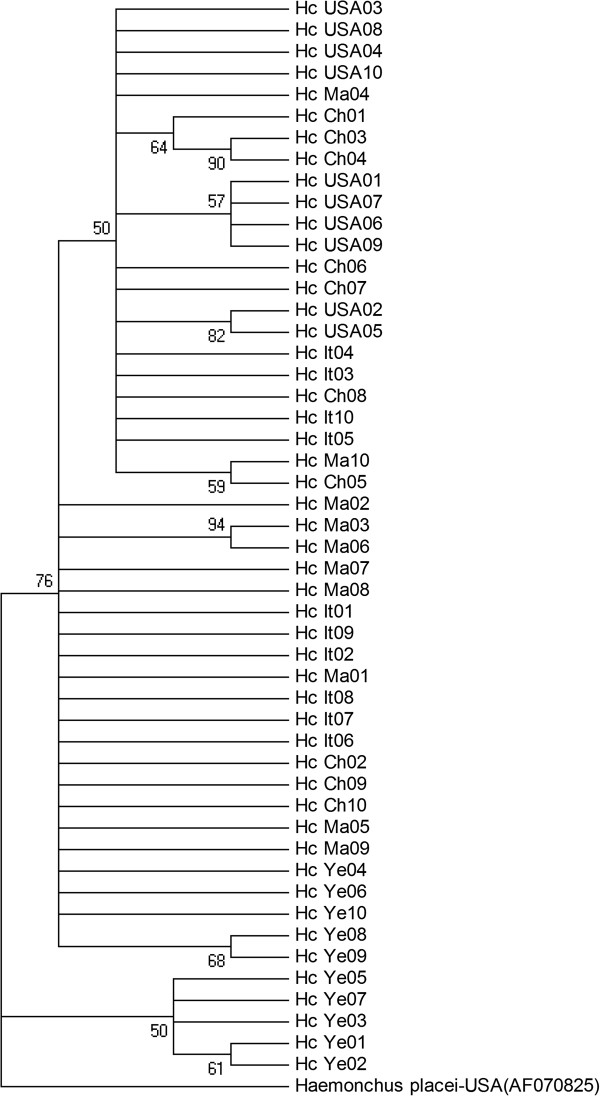
**Phylogenetic tree constructed using 50 *nad*4 sequences from *Haemonchus contortus *from five different countries.** The bootstrap values of >50% were displayed in the tree. Forty *nad*4 sequences of *H. contortus* were retrieved from the GenBank database and used for comparison; ten sequences represented each country: the USA (accession nos. AF070736-AF070745 [5]); Italy (AJ429793-AJ429802 [8]); Malaysia and Yemen (HQ660353-HQ660362 and HQ660269-HQ660278 [9]). *Haemonchus placei* (accession no. AF070785) was used as the outgroup. Abbreviations: Ch: China; It: Italy; Ma: Malaysia; USA: United States of America; Ye: Yemen.

In order to genetically compare *H. contortus* from China with those from other parts of the world, according to the approach used by Troell *et al.* (2006) [7], ten *nad*4 sequences, chosen randomly from the Chinese sample set and from each of the four other *H. contortus* populations representing other countries (Italy, Malaysia, the USA and Yemen) were included (Figure 3). The three methods produced consensus trees, in which there was no clear grouping according to country (at pp > 80%). In contrast, on three occasions, two samples from the same country (two each from China, Malaysia and the USA) grouped together with strong nodal support (pp > 80%). In addition, five samples from Yemen grouped together with low support (50%).

### Population genetic structure

The random distribution of 142 *nad*4 haplotypes from Chinese samples across the parsimony network (Figure 2) did not support any particular genetic structure among *H. contortus* specimens, which is corroborated by the low pairwise Fst/Nst values (Table 5), despite that they originated from different geographical locations. Although Fst and Nst were usually low between populations, ranging from -0.00005 to 0.19774 for Fst and -0.00332 to 0.20091 for Nst, the Yunnan population showed the highest level of genetic differentiation from other populations in China, with Fst values ranging from 0.08288 to 0.19774 and Nst values from 0.08386 to 0.20091 (Table 5).

**Table 5:** **Pairwise Fst and Nst values between *Haemonchus contortus *populations from seven different geographical locations in China, calculated from the sequences of the *nad *4 haplotypes**

**Location**	**SZ**	**YD**	**GX**	**YN**	**SX**	**LN**	**HLJ**
SZ		0.04043	0.00706	0.14808	0.03015	0.03587	-0.00328
YD	0.04088		0.06536	0.13184	0.05211	0.06440	0.03684
GX	0.00730	0.06593		0.11628	0.06263	0.00652	0.02926
YN	0.15081	0.13427	0.11799		0.19774	0.08288	0.17365
SX	0.03038	0.05293	0.06350	0.20091		0.06595	-0.00005
LN	0.03679	0.06501	0.00690	0.08386	0.06712		0.04311
HLJ	-0.00332	0.03735	0.03007	0.17688	-0.00016	0.04426	

Locations: Suizhou (SZ), Yidu (YD), Guangxi (GX), Yunnan (YN), Shaanxi (SX), Liaoning (LN) and Heilongjiang (HLJ). Fst and Nst are above and below the diagonal, respectively. Negative values indicate that more nucleotide substitutions occur within than between populations.

To assess possible factors that might affect gene flow, an analysis of molecular variance (AMOVA) was computed among populations. With the introduction of three-levels in the analysis, it was shown that 92.4% of the variance was distributed within populations and only 7.6% among populations (Table 6). The average Fst within seven populations (0.0759) was greatest compared with an Fst value among groups of 0.0143, and an Fst among populations within groups of 0.0655.

**Table 6:** **Analysis of Molecular Variance (AMOVA) for seven populations of *Haemonchus contortus *from China**

**Variance component**	**Variance**	**% of total**	**P**	**F-statistic**
Among populations	0.4007	7.59		
Within populations	4.8814	92.41	0.00^b^	Fst = 0.0759
Among groups^a^	0.0759	1.43	0.32	Fct = 0.0143
Within groups^a^	0.3423	6.46	0.00^b^	Fsc = 0.0655

^a^The populations from the six provinces were divided into three groups according to their geographical origins, including Southwest (Guangxi and Yunnan), Central (Suizhou, Yidu and Shaanxi) and Northeast (Liaoning and Heilongjiang) in China.

^b^*P* < 0.05.

Fst values were also calculated between samples from China and those from each of the other four countries, including Italy, Malaysia, the USA and Yemen, for which *nad*4 sequence data were available. The results revealed that the highest level of genetic differentiation was recorded between China and the USA, with an Fst value of 0.41294 (Table 2). Moderate levels of genetic differentiation were recorded between China and Italy, or China and Yemen, with the Fst values of 0.14407 and 0.26710, respectively. The lowest level of differentiation was found between China and Malaysia, with an Fst value of 0.05841 (Table 2).

## Discussion

In the present study, the ITS-2 and partial *nad*4 sequences were determined from 152 individual *H. contortus* specimens from seven populations of China. The ITS-2 sequences from these worms confirmed their specific identity as *H. contortus*. Further analysis revealed sequence variation of 2.6% in ITS-2 among all 152 individual worms from all locations in China. This magnitude of variation is consistent with variation (2.6%) detected in *H. contortus* populations in various countries, such as Germany and Sweden/Kenya [25,26], but lower than that (5.2%) detected between or among populations from seven countries, including Australia, France, Germany, New Zealand, Switzerland, The Netherlands and the UK [11]. For *nad*4, the nucleotide diversities within each of the seven *H. contortus* populations in China ranged from 0.018 to 0.037, with an average nucleotide diversity of 0.027 (Tables 2 and 4), which is in accordance with previously published data for this mitochondrial gene in various countries including Italy (0.026-0.03), Malaysia (0.032-0.044), the USA (0.024-0.03) and Yemen (0.021-0.036) [5,8,10]. Similarly, the high degree of diversity (i.e. 142 distinct haplotypes representing 152 individuals) and a mean haplotype diversity of 0.996 were in accordance with previous studies of the trichostrongyloids, such as *Haemonchus placei* and *Teladorsagia circumcincta*[7,27,28].

The present phylogenetic analysis revealed that there was no clear grouping of *H. contortus* according to host species (Additional file 2: Figure S1), a finding supported by the lack of significant differences in pairwise Fst and Nst values between *H*. *contortus* populations from LN or HLJ (sheep) and those from SZ, YD, GX, YN and SX (goat) (see Table 5). This result is similar to findings of previous studies, showing a limited relationship of *H. contortus* populations with different ruminant species in Brazil, Italy, Malaysia and Yemen [8-10].

The genetic analysis of the *nad*4 gene in the present study showed that the majority (92.4%) of genetic diversity was partitioned within populations of *H*. *contortus* from China, with no clear phylo-geographic structuring (with the exception of a subset of seven specimens from Yunnan), suggesting a high gene flow without clear geographical barriers among populations from different provinces. This proposal was supported by evidence of low pairwise Fst values between Chinese populations. The greatest nucleotide diversity within the *H. contortus* population from Yunnan, and the highest Fst values between this population and other Chinese populations, might relate to the fact that this province is particularly mountainous throughout, possibly preventing dispersal and gene flow.

On the other hand, on a global scale, the highest Fst value between *H. contortus* from China and the USA indicated a high genetic differentiation and less gene flow between these populations from two distinct continents. In contrast, the lowest genetic differentiation of Chinese specimens from those from Malaysia was reflected in the lowest pairwise Fst value. These results support a previous proposal that the global population genetic structure of *H. contortus* is characterized by no or low genetic differentiation between specimens from abutting geographical regions or within continents with limited barriers to gene flow, but significant genetic differentiation on a global scale (across continents) with substantial barriers to gene flow [7].

Previous studies have attributed a high degree of sequence diversity in the *nad*4 within populations of *H. contortus* to a large effective population size (Ne), high biotic potential, rapid, direct life-cycle and a high mutation rate in this polymorphic nematode [6,9,29], which explains the population genetic characteristics in China. In this country, haemonchosis is widespread geographically, and the prevalence and intensity of *H. contortus* infection are relatively high. An appraisal of key studies published in Chinese [30-34] shows that *H*. *contortus* exists in at least 30 of the 32 provinces in China, and is the dominant intestinal parasitic nematode of sheep and goats, with prevalences ranging from 6.5% to 100% and infection intensities of at least 20–8000 worms per host. The movement of livestock throughout China is also common. Since 1978, the livestock industry has undergone an enormous transformation. By the end of 1996, the total numbers of goats and sheep reached 303 million head, and the number has increased by 78.5% since that time, with a high demand on the red meat market for lamb [35]. Taken together, these findings indicate that the population genetic characteristics of *H. contortus* in China appear to be similar to those in other countries and studies.

As there is no commercial vaccine against haemonchosis in China, like elsewhere, the control of *H. contortus* relies largely on anthelmintics [36]. Here, the most widely used anthelmintics are albendazole, mebendazole, levamisole and ivermectin [36]. In spite of the apparently excessive use of anthelmintics against trichostrongyloids [36], no precise information is available on the nature and extent of drug resistance in *H. contortus* in China. Nonetheless, there are a small number of studies [37-39] reporting drug resistance against benzimidazoles and ivermectin in *H. contortus* populations in China. As the level of gene flow among populations of *H. contortus* parasitizing goats and sheep in China was shown here to be very high, there is substantial opportunity for rare resistance alleles to spread, indicating an urgent need to conduct a nationwide investigation of drug resistance in *H. contortus*. Such a focus will inform the scientific and non-scientific communities about the prevalence and intensity of resistance, and should underpin future control efforts in China.

## Conclusions

In conclusion, this is the first study of genetic variation within *H*. *contortus* in China. The results revealed high within-population variation, low population genetic differentiation and high gene flow of *H. contortus* in China. The results also indicated that on a global scale, there is no or a low level of genetic differentiation among *H. contortus* populations from the same continent, and significantly higher levels of genetic differentiation among those from different continents. These findings have important implications for studying the molecular epidemiology and controlling the spread of anthelmintic resistance against *H. contortus* in China.

## Abbreviations

AMOVA: Analysis of molecular variance; ITS-2: The second internal transcribed spacer of ribosomal DNA; L3s: The third-stage larvae; MJ: Median joining; ML: Maximum likelihood; MP: Maximum parsimony; nad4: Mitochondrial nicotinamide dehydrogenase subunit 4 gene; Ne: A large effective population size; NJ: Neighbour-joining; PCR: Polymerase chain reaction.

## Competing interests

The authors declare that they have no competing interests.

## Authors’ contributions

MH conceived the project. MB, WH, FZ, GZ, CW, XY, YZ, JZ, RF collected samples. FY and FL carried out laboratory work. FY performed the data analyses. FY and MH interpreted the data. FY, RBG and MH wrote the manuscript. All authors read and approved the final manuscript.

## Supplementary Material

Additional file 1 **Alignment of the 18 unique ITS-2 sequence types representing 152 individual adults of *Haemonchus contortus *from seven different geographical locations in China.** The accession number for each sequence is given in Table 3. Click here for file

Additional file 2 **The neighbor-joining (NJ) tree displaying the relationship among the 142 *nad*4 sequence types representing 152 individual adults of *Haemonchus contortus *from seven different geographical locations in China.** Each terminal branch represents one sequence. Each individual sequence is labeled according to geographical origin of the worm from which it was derived. Bootstrap values of >50% are indicated above or below the branches. *Haemonchus placei* (accession no. AF70785) was used as the outgroup. Click here for file
