# Supplementary material for: Genetic variability within and among Haemonchus contortus isolates from goats and sheep in China
Source: Parasit Vectors. 2013 Sep 25;6:279. doi: 10.1186/1756-3305-6-279 (PMC3852563; doi:10.1186/1756-3305-6-279)
Supplement: Additional file 1 — Alignment of the 18 unique ITS-2 sequence types representing 152 individual adults of Haemonchus contortus from seven different geographical locations in China. The accession number for each sequence is given in Table 3. [file 1756-3305-6-279-S1.doc]

**Additional file 1**

Ln9 AACCATATACTACAATGTGGCCAATTTCAACATTGTTTGTCAAATGGCATTTGTCTTTTA 60

Sz21 AACCATATACTACAATGTGGCCAATTTCAACATTGTTTGTCAAATGGCATTTGTCTTTTA 60

Sz22 AACCATATACTACAATGTGGGCAATTTCAACATTGTTTGTCAAATGGCATTTGTCTTTTA 60

Sx7 AACCATATACTACAATGTGGGCAATTTCAACATTGTTTGTCAAATGGCATTTGTCTTTTA 60

X78803 AACCATATACTACAATGTGGCTAATTTCAACATTGTTTGTCAAATGGCATTTGTCTTTTA 60

SZ9 AACCATATACTACAATGTGGCTAATTTCAACATTGTTTGTCAAATGGCATTTGTCTTTTA 60

Hlj10 AACCATATACTACAATGTGGCTAATTTCAACATTGTTTGTCAAATGGCATTTGTCTTTTA 60

Sz13 AACCATATAATACAATGTGGGTAATTTCAACATTGTTTGTCAAATGGCATTTGTCTTTTA 60

Yn16 AACCATATAATACAATGTGGGTAATTTCAACATTGTTTGTCAAATGGCATTTGTCTTTTA 60

Yd2 AACCATATAATACAATGTGGCTAATTTCAACATTGTTTGTCAAATGGCATTTGTCTTTTA 60

Yd14 AACCATATAATACAATGTGGCTAATTTCAACATTGTTTGTCAAATGGCATTTGTCTTTTA 60

SZ18 AACCATATACTACAATGTGGGTAATTTCAACATTGTTTGTCAAATGGCATTTGTCTTTTA 60

YD7 AACCATATACTACAATGTGGGTAATTTCAACATTGTTTGTCAAATGGCATTTGTCTTTTA 60

Sz12 AACCATATACTACAATGAGGGCAATTTCAACATTGTTTGTCAAATGGCATTTGTCTTTTA 60

Ln1 AACCATATACTACAATGAGGGCAATTTCAACATTGTTTGTCAAATGGCATTTGTCTTTTA 60

Gx3 AACCATATACTACAATGAGGGCAATTTCAACATTGTTTGTCAAATGGCATTTGTCTTTTA 60

Sz1 AACCATATACTACAATGAGGGCAATTTCAACATTGTTTGTCAAATGGCATTTGTCTTTTA 60

Yn20 AACCATATACTACAATGAGGGTAATTTCAACATTGTTTGTCAAATGGCATTTGTCTTTTA 60

Gx7 AACCATATACTACAATGAGGGTAATTTCAACATTGTTTGTCAAATGGCATTTGTCTTTTA 60

********* ******* ** **************************************

Ln9 GACAATTCCCATTTCAGTTCAAGAACATATACATGCAACGTGATGTTATGAAATTGTAAC 120

Sz21 GACAATTCCCATTTCAGTTCAAGAACATATACATGCAACGTGATGTTATGAAATTGTAAC 120

Sz22 GACAATTCCCATTTCAGTTCAAGAACATATACATGCAACGTGATGTTATGAAATTGTAAC 120

Sx7 GACAATTCCCATTTCAGTTCAAGAACATATACATGCAACGTGATGTTATGAAATTGTAAC 120

X78803 GACAATTCCCATTTCAGTTCAAGAACATATACATGCAACGTGATGTTATGAAATTGTAAC 120

SZ9 GACAATTCCCATTTCAGTTCAAGAACATATACATGCAACGTGATGTTATGAAATTGTAAC 120

Hlj10 GACAATTCCCATTTCAGTTCAAGAACATATACATGCAACGTGATGTTATGAAATTGTAAC 120

Sz13 GACAATTCCCATTTCAGTTCAAGAACATATACATGCAACGTGATGTTATGAAATTGTAAC 120

Yn16 GACAATTCCCATTTCAGTTCAAGAACATATACATGCAACGTGATGTTATGAAATTGTAAC 120

Yd2 GACAATTCCCATTTCAGTTCAAGAACATATACATGCAACGTGATGTTATGAAATTGTAAC 120

Yd14 GACAATTCCCATTTCAGTTCAAGAACATATACATGCAACGTGATGTTATGAAATTGTAAC 120

SZ18 GACAATTCCCATTTCAGTTCAAGAACATATACATGCAACGTGATGTTATGAAATTGTAAC 120

YD7 GACAATTCCCATTTCAGTTCAAGAACATATACATGCAACGTGATGTTATGAAATTGTAAC 120

Sz12 GACAATTCCCATTTCAGTTCAAGAACATATACATGCAACGTGATGTTATGAAATTGTAAC 120

Ln1 GACAATTCCCATTTCAGTTCAAGAACATATACATGCAACGTGATGTTATGAAATTGTAAC 120

Gx3 GACAATTCCCATTTCAGTTCAAGAACATATACATGCAACGTGATGTTATGAAATTGTAAC 120

Sz1 GACAATTCCCATTTCAGTTCAAGAACATATACATGCAACGTGATGTTATGAAATTGTAAC 120

Yn20 GACAATTCCCATTTCAGTTCAAGAACATATACATGCAACGTGATGTTATGAAATTGTAAC 120

Gx7 GACAATTCCCATTTCAGTTCAAGAACATATACATGCAACGTGATGTTATGAAATTGTAAC 120

************************************************************

Ln9 ATTCCTGAATGATATGAACATGTTGCCACTATTTGAGTGTACTCAGCGAATATTGAGATT 180

Sz21 ATTCCTGAATGATATGAACATGTTGCCACTATTTGAGTGTACTCAGCGAATATTGAGATT 180

Sz22 ATTCCTGAATGATATGAACATGTTGCCACTATTTGAGTGTACTCAGCGAATATTGAGATT 180

Sx7 ATTCCTGAATGATATGAACATGTTGCCACTATTTGAGTGTACTCAGCGAATATTGAGATT 180

X78803 ATCCCTGAATGATATGAACATGTTGCCACTATTTGAGTGTACTCAGCGAATATTGAGATT 180

SZ9 ATTCCTGAATGATATGAACATGTTGCCACTATTTGAGTGTACTCAGCGAATATTGAGATT 180

Hlj10 ATTCCTGAATGATATGAACATGTTGCCACTATTTGAGTGTACTCAGCGAATATTGAGATT 180

Sz13 ATTCCTGAATGATATGAACATGTTGCCACTATTTGAGTGTACTCAGCGAATATTGAGATT 180

Yn16 ATTCCTGAATGATATGAACATGTTGCCACTATTTGAGTGTACTCAGCGAATATTGAGATT 180

Yd2 ATTCCTGAATGATATGAACATGTTGCCACTATTTGAGTGTACTCAGCGAATATTGAGATT 180

Yd14 ATTCCTGAATGATATGAACATGTTGCCACTATTTGAGTGTACTCAGCGAATATTGAGATT 180

SZ18 ATTCCTGAATGATATGAACATGTTGCCACTATTTGAGTGTACTCAGCGAATATTGAGATT 180

YD7 ATTCCTGAATGATATGAACATGTTGCCACTATTTGAGTGTACTCAGCGAATATTGAGATT 180

Sz12 ATCCCTGAATGATATGAACATGTTGCCACTATTTGAGTGTACTCAGCGAATATTGAGATT 180

Ln1 ATCCCTGAATGATATGAACATGTTGCCACTATTTGAGTGTACTCAGCGAATATTGAGATT 180

Gx3 ATTCCTGAATGATATGAACATGTTGCCACTATTTGAGTGTACTCAGCGAATATTGAGATT 180

Sz1 ATTCCTGAATGATATGAACATGTTGCCACTATTTGAGTGTACTCAGCGAATATTGAGATT 180

Yn20 ATTCCTGAATGATATGAACATGTTGCCACTATTTGAGTGTACTCAGCGAATATTGAGATT 180

Gx7 ATTCCTGAATGATATGAACATGTTGCCACTATTTGAGTGTACTCAGCGAATATTGAGATT 180

** *********************************************************

Ln9 GACTTAGATAGTGACTTGTATGGCGACGATGTTCTTTTATCATTTGTATAA 231

Sz21 GACTTAGATAGTGACATGTATGGCGACGATGTTCTTTTATCATTTGTATAA 231

Sz22 GACTTAGATAGTGACATGTATGGCGACGATGTTCTTTTATCATTTGTATAA 231

Sx7 GACTTAGATAGTGACTTGTATGGCGACGATGTTCTTTTATCATTTGTATAA 231

X78803 GACTTAGATAGTGACATGTATGGCGACGATGTTCTTTTATCATTTGTATAA 231

SZ9 GACTTAGATAGTGACATGTATGGCGACGATGTTCTTTTATCATTTGTATAA 231

Hlj10 GACTTAGATAGTGACTTGTATGGCGACGATGTTCTTTTATCATTTGTATAA 231

Sz13 GACTTAGATAGTGACATGTATGGCGACGATGTTCTTTTATCATTTGTATAA 231

Yn16 GACTTAGATAGTGACTTGTATGGCGACGATGTTCTTTTATCATTTGTATAA 231

Yd2 GACTTAGATAGTGACATGTATGGCGACGATGTTCTTTTATCATTTGTATAA 231

Yd14 GACTTAGATAGTGACTTGTATGGCGACGATGTTCTTTTATCATTTGTATAA 231

SZ18 GACTTAGATAGTGACTTGTATGGCGACGATGTTCTTTTATCATTTGTATAA 231

YD7 GACTTAGATAGTGACATGTATGGCGACGATGTTCTTTTATCATTTGTATAA 231

Sz12 GACTTAGATAGTGACTTGTATGGCGACGATGTTCTTTTATCATTTGTATAA 231

Ln1 GACTTAGATAGTGACATGTATGGCGACGATGTTCTTTTATCATTTGTATAA 231

Gx3 GACTTAGATAGTGACTTGTATGGCGACGATGTTCTTTTATCATTTGTATAA 231

Sz1 GACTTAGATAGTGACATGTATGGCGACGATGTTCTTTTATCATTTGTATAA 231

Yn20 GACTTAGATAGTGACATGTATGGCGACGATGTTCTTTTATCATTTGTATAA 231

Gx7 GACTTAGATAGTGACTTGTATGGCGACGATGTTCTTTTATCATTTGTATAA 231

*************** ***********************************
