# Supplementary figures and images for: Genetic variability within and among Haemonchus contortus isolates from goats and sheep in China
Source: Parasit Vectors. 2013 Sep 25;6:279. doi: 10.1186/1756-3305-6-279 (PMC3852563; doi:10.1186/1756-3305-6-279)

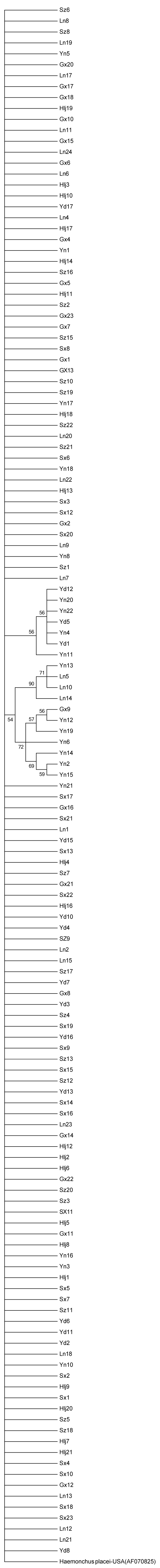

Supplement: Additional file 2 — The neighbor-joining (NJ) tree displaying the relationship among the 142 nad4 sequence types representing 152 individual adults of Haemonchus contortus from seven different geographical locations in China. Each terminal branch represents one sequence. Each individual sequence is labeled according to geographical origin of the worm from which it was derived. Bootstrap values of >50% are indicated above or below the branches. Haemonchus placei (accession no. AF70785) was used as the outgroup. [file 1756-3305-6-279-S2.tiff]
